# Akzeptanz einer telemetrischen Versorgung bei Patienten mit sICD-Sicherheitshinweis: Qualitative Interviewstudie der kardiologischen Versorgungsforschung

**DOI:** 10.1007/s00399-023-00938-3

**Published:** 2023-04-27

**Authors:** Leonie König, Elisabeth Grünberg, Panagiotis Xynogalos, Dierk Thomas, Rasmus Rivinius, Norbert Frey, Charlotte Ullrich, Ann-Kathrin Rahm

**Affiliations:** 1 Allgemeinmedizin und Versorgungsforschung, Universitätsklinikum Heidelberg, Heidelberg, Deutschland; 2 Stabstelle Versorgungsentwicklung und -qualität, kbo – Kliniken des Bezirks Oberbayern, München, Deutschland; 3 Kardiologie, Angiologie, Pneumologie, Universitätsklinikum Heidelberg, Heidelberg, Deutschland; 4 Heidelberger Zentrum für Herzrhythmusstörungen, Universitätsklinikum Heidelberg, Heidelberg, Deutschland; 5 DZHK (Deutsches Zentrum für Herz-Kreislauf-Forschung), Partnerstandorte Heidelberg/Mannheim, Heidelberg/Mannheim, Deutschland

**Keywords:** Telemedizin, Telemonitoring, ICD-Nachsorge, Qualitative Forschung, Versorgungsforschung, Telemedicine, Telemonitoring, Implantable cardioverter–defibrillator follow-up, Qualitative research, Health services research

## Abstract

**Hintergrund:**

Telemonitoring dient der Überwachung von implantierbaren Kardioverter-Defibrillatoren (ICD). Trotz der wissenschaftlich nachgewiesenen Effektivität und Sicherheit der telemetrischen Versorgung zeigen Studien, dass das Angebot nicht bei allen Patienten mit der Nutzung und Akzeptanz des Telemonitorings einhergeht.

**Fragestellung:**

Ziel dieses Forschungsvorhabens ist die Untersuchung der Einstellungen von ICD-Patienten zum Telemonitoring. Dabei ist von Interesse, welche Aspekte Einfluss auf die Haltung und Entscheidungsfindung nehmen.

**Methode:**

Die Datenerhebung erfolgte anhand teilstrukturierter leitfadengestützter Einzelinterviews. Es wurden insgesamt 14 Patienten mit subkutanem ICD und sowohl primär als auch sekundärprophylaktischer Indikation rekrutiert. Die Datenauswertung erfolgte nach inhaltlich strukturierender qualitativer Vorgehensweise.

**Ergebnis:**

Die Studie hat gezeigt, dass die Patienten mit Telemonitoring einen hohen Nutzen sehen, bei geringem Bedenken hinsichtlich der digitalen Technik. Bei Patienten ohne Telemonitoring verhält es sich umgekehrt. Die medizinischen Vorerfahrungen der Patienten haben einen wesentlichen Einfluss auf die Akzeptanz des Telemonitorings. Die technische Umsetzung und die praktische Handhabung des Telemonitorings wurden von Patienten aller Altersgruppen als einfach und unkompliziert angesehen.

**Diskussion:**

Die Ergebnisse lassen darauf schließen, dass die primär- und sekundärprophylaktische Indikation für eine ICD-Implantation einen Einfluss auf die Einstellung zum Telemonitoring und somit auf dessen Akzeptanz haben. Die Ergebnisse geben Anlass zu weiterer qualitativer Forschung im Hinblick auf die Nutzerakzeptanz zum Telemonitoring anderer ICD-Systeme.

## Abstract (en)

**Background:**

Telemonitoring is used to monitor implantable cardioverter defibrillators (ICDs). Despite the scientifically proven effectiveness and safety of telemetric care, studies show that the offer is not used and accepted by all patients.

**Objectives:**

The aim of this study is to investigate the attitudes of ICD patients towards telemonitoring, including which aspects influence attitudes and decision-making.

**Methods:**

Data were collected using semi-structured, guideline-based individual interviews. A total of 14 patients with a subcutaneous ICD (sICD) and both primary and secondary prophylactic indications were recruited. Data analysis followed a content-structuring qualitative approach.

**Results:**

Patients with telemonitoring perceived a high benefit with low concerns about digital technology, while the opposite was observed for patients without telemonitoring. The patients’ previous medical experience has a crucial impact on the acceptance of telemonitoring. All age groups reported the technical implementation and practical handling of telemonitoring to be simple and uncomplicated.

**Conclusion:**

The results suggest that the primary and secondary prophylactic indication for ICD implantation have an influence on the attitude towards telemonitoring and, thus, on acceptance. Further qualitative research regarding user acceptance of telemonitoring of other ICD systems is needed.

## Hintergrund

Patienten können nach Implantation eines Kardioverter-Defibrillators (ICD) mittels Telemonitoring nachgesorgt werden [23, 25]. Trotz wissenschaftlich bewiesener Vorteile, wie Effektivität und Sicherheit, gibt es Patientengruppen, die eine direkte Nachsorge in Präsenz durch die Ärzte bevorzugen [7, 13–15, 22, 24]. Da die Nutzung des kardiologischen Telemonitorings in Zukunft zunehmen wird [9], ist es von Bedeutung, die Versorgung patientenorientiert zu gestalten sowie die Akzeptanz des Telemonitorings zu steigern [6, 10]. Ziel dieser Studie ist daher die Untersuchung der Einstellungen zum Telemonitoring von Patienten mit einem subkutanen Defibrillator und einem Sicherheitshinweis.

### Telemonitoring

Das Telemonitoring stellt eine Möglichkeit dar, mittels digitaler Technik, sowohl patientenspezifische Parameter als auch gerätespezifische Daten kontinuierlich zu erheben und an ein Überwachungszentrum zu übermittelt [3]. Dabei kann der Zeitraum zwischen dem Auftreten technischer oder medizinischer Störungen und dem Ergreifen entsprechender Maßnahmen verringert werden [21].

### Studienkontext

Im Rahmen der Studie wurde die Akzeptanz zum Telemonitoring von subkutanen ICD (sICD) der Firma Boston Scientific, Marlborough, Massachusetts, United States of America untersucht. Diese Firma stellt die telemetrische Nachsorge mittels des LATITUDE™ NXT WAVE Communicator zur Verfügung [5]. Hierfür aktiviert der Patient den Communicator und führt die Datenübertragung zu einem festgelegten Zeitraum eigenständig zuhause durch. Der Communicator überprüft den sICD und sammelt die Daten. Diese werden dann automatisch an eine gesicherte Internetseite gesendet, worauf ausschließlich das medizinische Versorgungsteam Zugriff hat. Der Vorgang umfasst lediglich eine Datenabfrage, es werden weder Eingriffe in die Funktion noch Änderungen an dem Implantat vorgenommen [5]. Im Jahr 2020 gab die Firma Boston Scientific einen Sicherheitshinweis bekannt [4]. Dieser Sicherheitshinweis umfasste den sICD vom Typ EMBLEM A209 oder A2019 sowie die sICD-Elektrode vom TYP EMBLEM Modell 3501 und die Empfehlung zur Anbindung der Patienten an das Telemonitoring.

## Methode

Als Leitlinie für den Forschungsprozess wurde ein qualitatives Studiendesign gewählt. Darüber hinaus lässt sich die Studie als eine nichtinterventionelle, nichtkontrollierte und nichtrandomisierte monozentrische Querschnittstudie charakterisieren. Die Patienten wurden anhand von teilstandardisierten leitfadengestützten Einzelinterviews befragt. Für die Strukturierung in der Datenerhebung und Konzeption des Leitfadens wurde auf aktuelle Modelle der Technikakzeptanzforschung [6, 11, 12, 17] und praxisorientierten Vorannahmen zurückgegriffen. Zusätzlich wurde eine Literaturrecherche von Januar bis Ende März in medizinischen Datenbanken wie PubMed (Medline) durchgeführt. Hierbei dienten zuvor definierte Schlüsselbegriffe („telemonitoring“, „implantable cardioverter-defibrillator“, „acceptance digital technologies“) als Hilfsmittel. Der Leitfaden beinhaltet folgende Fragedimensionen: Erfahrungen mit dem telemetrischen Angebot, Erfahrungen mit der Nutzung des Telemonitorings, Einfluss von sozialen Ressourcen und Erwartungen sowie Haltung der Patienten.

Zur Beschreibung der Stichprobe wurden zusätzlich noch soziodemografische Daten erhoben. Die Interviews wurden zwischen August und September 2021 face-to-face durchgeführt. Zuvor lag eine Zustimmung der Ethikkommission anhand eines positiven Ethikvotums der Universität Heidelberg vor (S-573/2021).

Die Transkription und Auswertung der Tonaufnahmen der Interviews erfolgten anhand der computergestützten qualitativen Daten- und Textanalyse-Software MAXQDA (Version 20.3). Das Auswertungsverfahren orientiert sich an der inhaltlich strukturierenden qualitativen Inhaltsanalyse nach Udo Kuckartz (2018). Hierbei wird das transkribierte Datenmaterial anhand von Kategorien und Subkategorien inhaltlich strukturiert [16].

### Rekrutierung und Sampling

Der Rekrutierungszeitraum erstreckte sich von Juli bis September 2021. Ein Einschlusskriterium war die Implantation eines sICD aufgrund von primär- oder sekundärprophylaktischer Indikation. Zusätzlich wurden ausschließlich Patienten des Herstellers Boston Scientific eingeschlossen, die von dem Sicherheitshinweis betroffen sind. Um mögliche Unterschiede in der Einstellung der Studienteilnehmenden aufdecken zu können, wurden in dieser Arbeit zwei Gruppen, eine mit Telemonitoring und eine das Telemonitoring ablehnende Gruppe, untersucht. Für die Patienten mit Telemonitoring wurde eine Mindestdauer der Nutzung von 3 Monaten festgelegt. Darüber hinaus waren alle Personen mindestens 18 Jahre alt und einwilligungsfähig. Demgegenüber wurden Patienten mit schwerwiegenden psychischen Erkrankungen sowie unzureichenden Deutschkenntnissen von der Studienteilnahme ausgeschlossen [1, 8].

## Ergebnis

### Patientenpopulation

Insgesamt konnten 14 Patienten (7 = w; 7 = m) rekrutiert werden. Die Gruppe ohne Telemonitoring umfasste 5 Patienten (2 = w; 3 = m). Die Gruppe mit Telemonitoring enthielt 9 Patienten (5 = w; 4 = m). Das durchschnittliche Alter aller Teilnehmenden betrug zum Zeitpunkt der Interviews 53 Jahre (Min. = 25 Jahre; Max. = 70 Jahre). Die Zeitspanne zwischen der Defibrillator-Implantation bis zum Telemonitoring-Angebot lag im Durchschnitt in der Gruppe mit Telemonitoring bei ca. 20 Monaten (Min. = 2; Max. = 39) und in der Gruppe ohne Telemonitoring bei ca. 38 Monaten (Min. = 4; Max. = 71). In der Gruppe mit Telemonitoring lag die Dauer der einzelnen Interviews im Mittel bei 41 min (Min. = 21 min; Max. = 64 min). In der Gruppe ohne Telemonitoring umfassten die Interviews eine Spanne von 21 min (Min. = 13 min; Max. = 32 min; Tab. 1).

**Table 1:** 

	Patienten mit Telemonitoring (*n* = 9)	Patienten ohne Telemonitoring (*n* = 5)	Gesamt (*n* = 14)
Alter (Jahre)	52 (25–70)	54 (34–65)	53
Geschlecht (weiblich)	5	2	7
Geschlecht (männlich)	4	3	7
Ø Zeitraum von Implantation bis Angebot zum Telemonitoring (Monate)	20 (2–39)	38 (4–71)	26
Ø Zeitraum von Angebot zum Telemonitoring bis Interviewtermin (Monate)	5 (2–8)^a^	7 (6–8)	6

^a^Zeitraum der telemetrischen Nutzung

### Wahrnehmung krankheitsbedingter (Vor‑)Erfahrungen

Es stellt sich heraus, dass die Indikation und die Erfahrungen bezüglich des sICD sowie die Informationen über den Sicherheitshinweis des Herstellers Boston Scientific bei den Patienten Verunsicherung bezüglich des sICD auslösen. Als Indikation wurde überwiegend von einem plötzlichen Herzstillstand (Sekundärprophylaxe) sowie von Herzrhythmusstörungen (Primärprophylaxe) berichtet. Es wird deutlich, dass die Patientengruppe mit Telemonitoring und überwiegender sekundärprophylaktischer Indikation emotionaler auf die Defibrillator-Implantation reagierte als die Gruppe ohne Telemonitoring. Die Patientengruppe mit Telemonitoring berichtet hierbei eher von einem unerwarteten und schockierenden Ereignis, welches sie überfordert hat. Hierbei äußern sich die Interviewten beispielhaft wie folgt:* „Ich war überfordert. … Ich war vorher noch nie im Krankenhaus. Dann war ich da halt mit dem Herzstillstand und dann Defi-Operation, ach Gott, was kommt da auf mich zu …“ (B_11* *w, Pos. 64)*. Patienten ohne Telemonitoring äußern sich demgegenüber eher nüchtern, wie an folgender Aussage deutlich wird: *„… Gut fand ich es nicht, aber wie gesagt, so als, als Lebensversicherung hab’ ich’s halt dann doch machen lassen“ (B_10* *mN, Pos. 16).*

### Wahrnehmung des Informationsangebots

Im Hinblick auf die Informationsvermittlung bezüglich des Telemonitorings wird deutlich, dass die Patienten aus beiden Gruppen über wenige Kenntnisse zum Telemonitoring verfügen. In der Gruppe ohne Telemonitoring erinnern sich die Patienten nur noch teilweise an das Aufklärungsgespräch. Dabei stellt das Aufklärungsgespräch die Hauptinformationsquelle zum Telemonitoring für beide Patientengruppen dar. Zusätzlich lässt sich festhalten, dass das Angebot an Informationen während des Aufklärungsgesprächs in beiden Patientenkollektiven gering ist. *„…, die … Ärztin, die diese erste Untersuchung … vornahm, ... sagte das nebenbei, dass es eben auch diese Möglichkeit gibt ..., aber großartig viel mehr Informationen habe ich da eigentlich nicht bekommen“ (B_10* *mN, Pos. 62).*

### Einflussfaktoren auf die Entscheidung

In der Patientengruppe ohne Telemonitoring waren die Technikskepsis und der Datenschutz wesentliche Einflussfaktoren für eine Ablehnung des Telemonitorings. Es zeigt sich, dass Patienten aus dieser Gruppe weniger Vertrauen in technische Geräte haben. *„… Ich trau dem Kram nicht so“ (B_12* *mN, Pos. 20).* Im Hinblick auf den Datenschutz äußert sich ein Patient dahingehend, dass ihm keine hundertprozentige Sicherheit hinsichtlich der Weiterverarbeitung seiner Daten gewährleistet werden konnte. *„… kann man sich immer sicher sein, wo die Daten von einem hingelangen? Wo ist da die hundertprozentige Sicherheit? …“ (B_1* *mN, Pos. 34).*

In der Gruppe mit Telemonitoring konnte eine offene Haltung gegenüber digitalen Technologien sowie ein persönlicher Nutzen als relevante Einflussfaktoren für den Entscheidungsprozess festgestellt werden. Im Hinblick auf die Technikeinschätzung sehen die Patienten das Telemonitoring als Möglichkeit, die mit vielen Vorteilen verbunden ist. Hierbei ist der vorhandene Sicherheitshinweis ein zentraler Aspekt. Sie erwarten, dass sie sich sicherer fühlen durch die telemetrische Überwachung und der damit verbundenen Möglichkeit frühzeitige Fehlfunktionen zu erkennen. *„Also ich erhoffe mir einfach, dass das ein Vorteil ist, wenn wirklich was wäre und man vielleicht das nicht mitkriegt … Ich glaub das ist … einfach so ein Sicherheitsaspekt“ (B_3* *w, Pos. 92–94).*

Misstrauen gegenüber dem Datenschutz wird von den Patienten nicht geäußert. Das Thema wird von diesen Patienten kaum angesprochen. Im Einzelfall wird dieser Aspekt in dem Kontext als weniger relevant erachtet.

### Erfahrungen mit Telemonitoring

**Figure 1:**
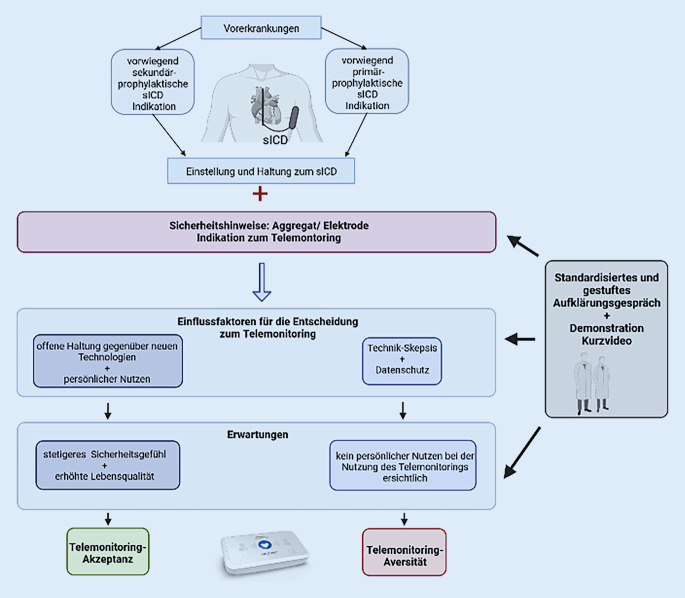

In diesem Punkt kann festgehalten werden, dass die Patienten überwiegend über positive Erfahrungen mit dem Telemonitoring berichten. Vor allem die Erwartungen hinsichtlich eines höheren Sicherheitsgefühls bestätigten sich. Der einfache Umgang und die leichte Installation des Communicators wurden von den Patienten dahingehend betont, dass die Nutzung der telemetrischen Versorgung sowie die Bedienung des Communicators auch für ältere Menschen geeignet sei. Darüber hinaus wird festgestellt, dass bei keinem der Patienten zum Zeitpunkt der Interviews bereits Auffälligkeiten durch die telemetrische Überwachung identifiziert worden sind (Abb. 1).

## Diskussion

Ein wesentlicher Aspekt für die Entscheidungsfindung ist die Indikation für die ICD-Implantation. Die Patienten mit vorwiegend sekundärprophylaktischer Indikation entschieden sich für das Telemonitoring. Diese Patienten verspüren eine größere Verunsicherung hinsichtlich ihres Gesundheitszustands als die Patienten mit primärprophylaktischer Indikation, die sich gegen das Telemonitoring entschieden [2]. Dies lässt vermuten, dass den Patienten mit Telemonitoring die Wichtigkeit der Funktionsweise des sICD bewusster ist als der Gruppe ohne Telemonitoring. Die Patienten ohne Telemonitoring scheinen die Notwendigkeit ihres Defibrillators als geringer einzuschätzen. Es scheint, dass die Patienten ohne Telemonitoring durch die geringere Verunsicherung keinen persönlichen Nutzen in der telemetrischen Überwachung sehen. Im Gegensatz dazu zeigen die Patienten mit Telemonitoring ein höheres Maß an Verunsicherung hinsichtlich ihres sICD. Sie haben die Erwartung, dass sie durch die telemetrische Versorgung und die kontinuierliche Überwachung ihres sICD ein stetigeres Sicherheitsgefühl im Alltag bekommen.

Es könnte geschlussfolgert werden, dass die Krankheitsvorerfahrungen die Einstellung zum implantierten Defibrillator prägen. Die Einstellung zum sICD könnte wiederum die Haltung und Akzeptanz zum Telemonitoring nachhaltig beeinflussen.

Zusätzlich zeigt sich, dass beide Patientengruppen Wissenslücken und Verständnisprobleme hinsichtlich der Funktionsweise des Telemonitorings haben. Vor allem bei den Patienten mit Telemonitoring scheint dies der Fall zu sein. Dabei zeigte sich, dass die Patienten sich weniger für die detaillierten technischen Aspekte der Funktionsweise des Telemonitorings interessieren, sondern vielmehr für die Auswirkungen des allgemeinen Erhalts bzw. der Verbesserung ihrer Lebensqualität. Dies steht im Einklang mit den Studienergebnissen der Untersuchung von Senn et al. (2020) [20].

Die Teilnehmenden sind sich mehrheitlich einig, dass es sich hierbei um eine einfache Nutzung handelt. Die Praktikabilität wird dahingehend betont, dass die Patienten der Meinung sind, dass die telemetrische Abfrage auch für ältere Menschen geeignet und bewältigbar sei. Da der ältere Bevölkerungsanteil den Großteil an Patienten für eine ICD-Implantation ausmacht [2], scheint das Telemonitoring auch für die Hauptanwendergruppe eine mögliche Versorgungsform zu sein. Es wird angenommen, dass die technischen Anforderungen für die Nutzung des Telemonitorings kein Hindernis für die Akzeptanz dieser Patientengruppe und somit für die Verbreitung in der kardiologischen Versorgung darstellen. Dies bestätigt auch den Forschungsstand zur Technikakzeptanz bei älteren Menschen [18, 19]. Zusätzlich stimmen die positiven Erfahrungswerte der Patienten mit den Ergebnissen der REMOTE-Studie zur hohen Patientenzufriedenheit hinsichtlich der Fernüberwachung überein [24].

## Limitationen

Ein längerer Erhebungszeitraum sowie eine multizentrische Analyse und andere Erhebungszeitpunkte könnten noch differenziertere Ergebnisse hervorbringen. Bei allen eingeschlossenen Patienten liegt ein Sicherheitshinweis vor. Zusätzlich umfasst die Stichprobe ausschließlich sICD-Patienten des Herstellers Boston Scientific.

## Fazit für die Praxis

- Im Aufklärungsgespräch sollte den Patienten mit primärprophylaktischer Indikation besondere Aufmerksamkeiten geschenkt werden.
- Auf Grund der mitunter geringer wahrgenommenen Notwendigkeit sollte die Nutzung der telemetrischen Versorgung umfassend erläutert werden.
- Ein standardisiertes, gestuftes Aufklärungsgespräch ggf. mit Anwendung von Kurzvideos zur Gerätedemonstration kann für ein qualitativ und quantitativ verbessertes Informationsangebot sorgen.
- Zudem kann die Demonstration des Communicators Unsicherheiten verringern.
